# Decongestive treatment adjustments in heart failure patients remotely monitored with a multiparametric implantable defibrillators algorithm

**DOI:** 10.1002/clc.23832

**Published:** 2022-05-03

**Authors:** Federico Guerra, Antonio D'Onofrio, Ermenegildo De Ruvo, Michele Manzo, Luca Santini, Giovanna Giubilato, Carmelo La Greca, Barbara Petracci, Giulia Stronati, Valter Bianchi, Annamaria Martino, Fabio Franculli, Paolo Compagnucci, Monica Campari, Sergio Valsecchi, Antonio Dello Russo

**Affiliations:** ^1^ Cardiology and Arrhythmology Clinic Marche Polytechnic University, “Ospedali Riuniti” Ancona Italy; ^2^ Unità Operativa di Elettrofisiologia, Studio e Terapia delle Aritmie, Monaldi Hospital Naples Italy; ^3^ Policlinico Casilino Rome Italy; ^4^ OO.RR. San Giovanni di Dio Ruggi d'Aragona Salerno Italy; ^5^ “Giovan Battista Grassi” Hospital Rome Italy; ^6^ “Spaziani” Hospital Frosinone Italy; ^7^ Fondazione Poliambulanza Brescia Italy; ^8^ IRCCS Policlinico San Matteo Pavia Italy; ^9^ Boston Scientific Italia Milan Italy

**Keywords:** CR0, decompensation, diuretics, heart failure, ICD, remote monitoring

## Abstract

**Aims:**

HeartLogic algorithm combines data from multiple implantable defibrillators (ICD)‐based sensors to predict impending heart failure (HF) decompensation. A treatment protocol to manage algorithm alerts is not yet known, although decongestive treatment adjustments are the most frequent alert‐triggered actions reported in clinical practice. We describe the implementation of HeartLogic for remote monitoring of HF patients, and we evaluate the approach to diuretic dosing and timing of the intervention in patients with device alerts.

**Methods:**

The algorithm was activated in 229 ICD patients at eight centers. The median follow‐up was 17 months (25th–75th percentile: 11–24). Remote data reviews and patient phone contacts were undertaken at the time of HeartLogic alerts, to assess the patient's status and to prevent HF worsening. We analyzed alert‐triggered augmented HF treatments, consisting of isolated increases in diuretics dosage.

**Results:**

We reported 242 alerts (0.8 alerts/patient‐year) in 123 patients, 137 (56%) alerts triggered clinical actions to treat HF. The HeartLogic index decreased after the 56 actions consisting of diuretics increase. Specifically, alerts resolved more quickly when the increases in dosing of diuretics were early rather than late: 28 days versus 62 days, *p* < .001. The need of hospitalization for further treatments to resolve the alert condition was associated with higher HeartLogic index values on the day of the diuretics increase (odds ratio: 1.11, 95% CI: 1.02–1.20, *p* = .013) and with late interventions (odds ratio: 5.11, 95% CI: 1.09–24.48, *p* = .041). No complications were reported after drug adjustments.

**Conclusions:**

Decongestive treatment adjustments triggered by alerts seem safe and effective. The early use of decongestive treatment and the use of high doses of diuretics seem to be associated with more favorable outcomes.

## 1 INTRODUCTION

Heart failure (HF) is one of the leading causes of hospital admission worldwide, and is associated with high morbidity, mortality, and rehospitalization.^1^ Remote monitoring and advanced diagnostic algorithms have been developed to provide continuous data on HF patients who receive implantable defibrillators (ICD) and resynchronization therapy (CRT‐D). Many studies have reported combining ICD diagnostics to better stratify and manage patients at risk of HF events,^2^, ^3^ and current guidelines suggest that ICD‐based multiparameter monitoring may be considered, to improve clinical outcomes.^1^ The HeartLogic (Boston Scientific) algorithm combines data from multiple ICD‐ and CRT‐D‐based sensors and has proved to be a sensitive and timely predictor of impending HF decompensation.^4^ Although its diagnostic performance in detecting HF worsening has been demonstrated, it is still not known what treatment protocol should be applied to manage the events notified by the algorithm.

Most of the symptoms associated with acute HF are the result of excessive fluid retention, and loop diuretics are the treatment of choice to combat them.^5^ Decongestive treatment adjustments not only constitute the most common intervention when patients are hospitalized for acute HF,^6^, ^7^ but were also reported to be the most frequently triggered actions in response to alerts in the first experiences of HeartLogic use in clinical practice.^8^, ^9^ The aim of the present study was to describe the implementation of the HeartLogic algorithm in a protocol for the remote monitoring of HF patients, and to evaluate the approach to diuretic dosing and timing of the intervention in patients with device alerts.

## 2 METHODS

The study was conducted in eight Italian high‐volume arrhythmia centers. HeartLogic was activated in all HF patients with reduced left ventricular ejection fraction (≤35% at the time of implantation) who had received a HeartLogic‐enabled ICD or CRT‐D device (RESONATE family, Boston Scientific) between December 2017 and July 2020, in accordance with standard indications,^1^ and were consecutively enrolled in the LATITUDE (Boston Scientific) remote monitoring platform. Patients were followed in accordance with the standard practice of the participating centers, based on current international recommendations.^10^ Clinics periodically checked the remote monitoring website for transmissions. Moreover, remote data reviews and patient phone contacts were undertaken at the time of HeartLogic alerts (when the index crossed the nominal threshold value of 16), to assess the patient's decompensation status and, if possible, to prevent further worsening. Data on the clinical events that occurred during follow‐up were collected at the study centers in the framework of a prospective registry. The study was carried out in accordance with institutional standards, national legal requirements, and the Declaration of Helsinki. The Institutional Review Boards approved the study (ClinicalTrials.gov identifier: NCT02275637), and all patients provided written informed consent for data storage and analysis.

### 2.1 HeartLogic index

The details of the HeartLogic algorithm have been reported previously.^4^ Briefly, the algorithm combines data from multiple sensors: accelerometer‐based first and third heart sounds, intrathoracic impedance, respiration rate, the ratio of respiration rate to tidal volume, night heart rate, and patient activity. Each day, the device calculates the sensor‐recorded values in terms of their shift from the baseline and computes a composite index. An alert is issued when this index crosses a programmable threshold. Weekly reminders (realerts) are sent until the HeartLogic index returns below the nominal alert recovery threshold.^5^

### 2.2 Alert management

The study protocol did not mandate any specific intervention algorithm, and physicians were free to remotely implement clinical actions (e.g., drug adjustments, educational interventions), to schedule extra in‐office visits when deemed necessary for additional investigations or for interventions, or to adopt an active monitoring approach. In our analysis, we classified the alerts according to the management strategy adopted at the centers. We distinguished between alerts followed/not followed by clinical actions, and analyzed alert‐triggered actions to treat HF (e.g., change in current HF medications, reinforcing adherence, device programming optimization). We then specifically investigated augmented HF treatments consisting of isolated increases in the equivalent dose of diuretics, as compared with the dose on the day before the initial alert. Increases in the dosing of diuretics were categorized as either early or late, according to when they were initiated: early treatments were those implemented within 2 weeks of the first alert notification (following the initial alert or the first weekly reminder), while late treatments were those undertaken after 2 weeks (following the second or subsequent weekly reminders). Diuretic increases were also categorized as either major or minor. Administering >2 times the daily dose of loop diuretics or switching to a more bioavailable diuretic were considered major actions,^11^ while lower increases were considered minor.

A shorter “in‐alert” state duration was considered suggestive of the efficacy of the intervention and of its ability to resolve the alert condition without requiring further treatments.

### 2.3 Statistical analysis

Descriptive statistics are reported as mean ± SD for normally distributed continuous variables, or medians with 25th to 75th percentiles in the case of nonnormal distributions. Normality of distribution was tested by means of the Kolmogorov–Smirnov test. The time course of HeartLogic index and sensor changes surrounding the decongestive treatment adjustment were evaluated at four time‐points, as in a previous study.^12^ A 30‐day baseline was compared both with a 7‐day preaction state measured up until the day before the diuretic augmentation, and with the state on the first day of the augmented therapy. Moreover, recovery was evaluated by recording sensor values over a 2‐week period beginning 2 weeks after diuretic augmentation and comparing these with the baseline values. For control purposes, averaged sensor data were calculated in patients who did not have HF events and decongestive treatment adjustments during clinical follow‐up. These trends were aligned on a random day during the observation period. Sensor data were compared between different temporal periods by means of a paired *t*‐test. Differences in non‐Gaussian variables were tested by means of the Mann–Whitney nonparametric test. Univariable binary logistic regression analysis was utilized to evaluate the relationship between the need for further treatment to resolve the alert condition and baseline clinical or treatment variables. All variables displaying a statistically significant difference (*p* < .05) were included in a multivariable binary logistic regression analysis. A *p*‐value  < .05 (two‐tailed) was considered significant in all tests. All statistical analyses were performed by means of R: a language and environment for statistical computing (R Foundation for Statistical Computing).

## 3 RESULTS

From December 2017 to July 2020, HeartLogic was activated in 229 patients who had received an ICD or CRT‐D. Table 1 shows the baseline clinical variables of all patients. The median follow‐up was 17 months (25th–75th percentile: 11–24) (a total of 308 patient‐years).

**Table 1:** Demographics and baseline clinical parameters of the study population

Parameter	Total *N* = 229
Male gender, *n* (%)	171 (75)
Age, years	69 ± 11
Ischemic etiology, *n* (%)	125 (54)
Coronary artery disease	108 (47)
NYHA class	
− Class I, *n* (%) − Class II, *n* (%) − Class III, *n* (%) − Class IV, *n* (%)	13 (6) 101 (44) 108 (47) 7 (3)
LV ejection fraction (%)	30 ± 8
AF history, *n* (%)	91 (40)
Diabetes, *n* (%)	75 (33)
COPD, *n* (%)	47 (20)
Chronic kidney disease, *n* (%)	85 (37)
Hypertension, *n* (%)	153 (67)
β‐Blocker use, *n* (%)	204 (89)
ACE‐inhibitor/ARB/ARNI use, *n* (%)	198 (86)
Diuretic use, *n* (%)	207 (90)
Antiarrhythmic use, *n* (%)	191 (28)
Ivabradine use, *n* (%)	26 (11)
CRT device, *n* (%)	197 (86)
Primary prevention, *n* (%)	199 (87)

Abbreviations: AF, atrial fibrillation; ARB, angiotensin receptor blocker; ARNI, angiotensin receptor neprilysin inhibitor; COPD, chronic obstructive pulmonary disease; CRT, cardiac resynchronization therapy; LV, left ventricular; NYHA, New York Heart Association.

### 3.1 HeartLogic alerts and their management

The HeartLogic index crossed the threshold value 242 times (0.8 alerts/patient‐year) in 123 patients (up to six times per patient). The in‐alert state lasted a median of 42 days (25th–75th percentile: 25–60). The overall time in the alert state was 33 patient‐years (11% of the total observation period). Of the 242 alerts, 137 (56%) triggered clinical actions to treat HF, while the remaining 105 were not followed by HF therapy changes because they were judged nonactionable, unexplained, or associated with non‐HF‐related conditions. Of the 137 alert‐triggered actions, 56 consisted of decongestive treatment augmentations only (increase in the equivalent dose of diuretics or switch to a more bioavailable diuretic) and 81 were mixed interventions. These latter included: 26 diuretic changes, 50 nondiuretic HF medication changes, 25 patient counseling on therapeutic adherence, 7 device programming optimization, and/or cardioversion. A single action was performed in 54 alerts and two actions in 27 alerts.

**Figure 1:**
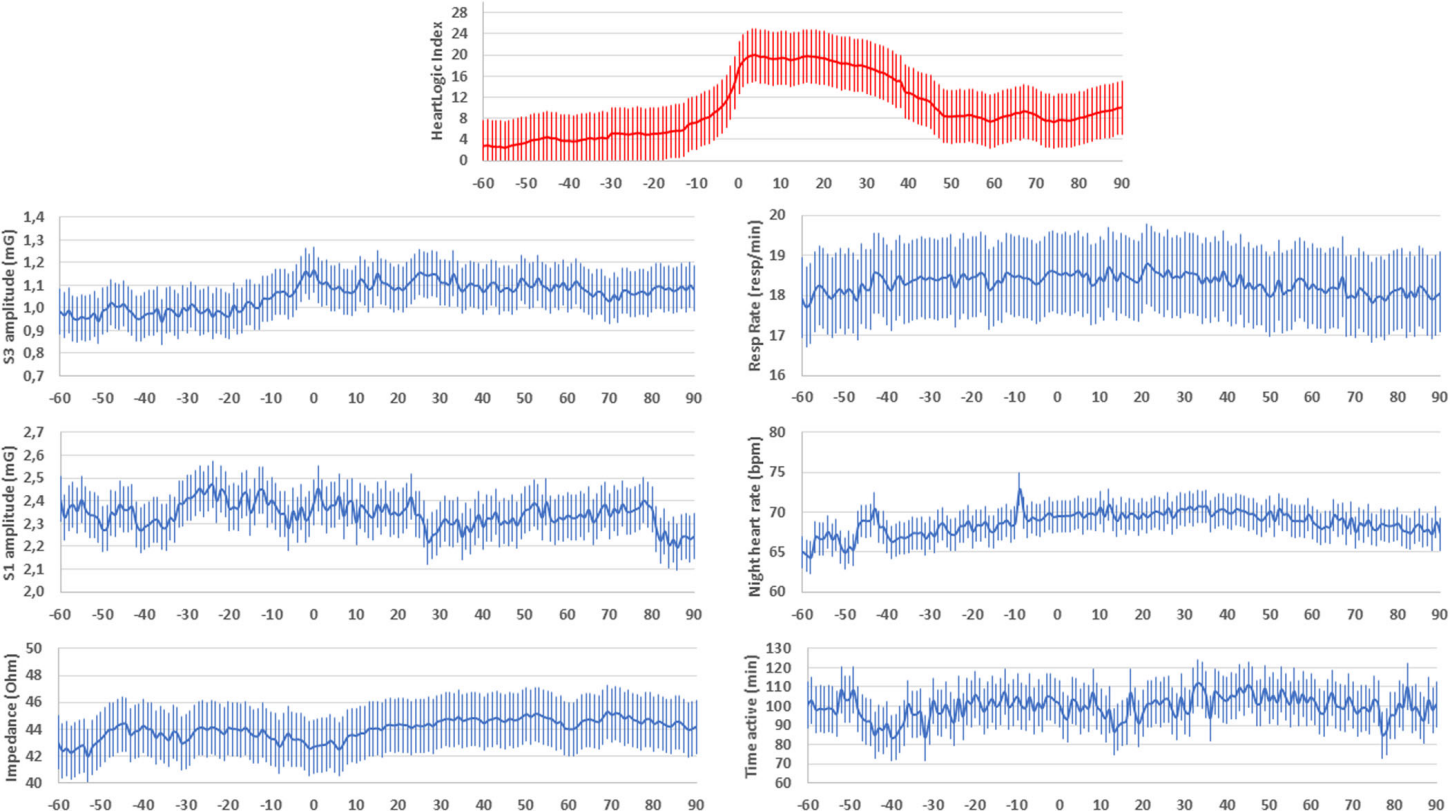
Average HeartLogic combined index and all physiologic parameters collected by the devices at the time of the alerts that triggered the 56 diuretic therapy adjustments (Day 0 is the day when the HeartLogic index crossed the threshold)

Figure 1 shows the average HeartLogic combined index, and all physiologic parameters collected by the devices at the time of the alerts that triggered the 56 diuretic therapy adjustments (Day 0 is the day when the HeartLogic index crossed the threshold). During the 30 days before the index exceeded the threshold value of 16, an increase in the amplitude of S3 can be noted, together with a slight decrease in S1 amplitude and an increase in the respiratory rate and night heart rate.

Of the 56 decongestive treatment adjustments, 30 were implemented within 2 weeks of the first alert notification (early actions—average time from alert to intervention 5 ± 4 days), while the remaining 26 took place later (late actions—average time 40 ± 27 days). In 29 cases, a major increase in diuretic therapy was noted, while in 27 cases the increase in the daily dose was minor.

**Figure 2:**
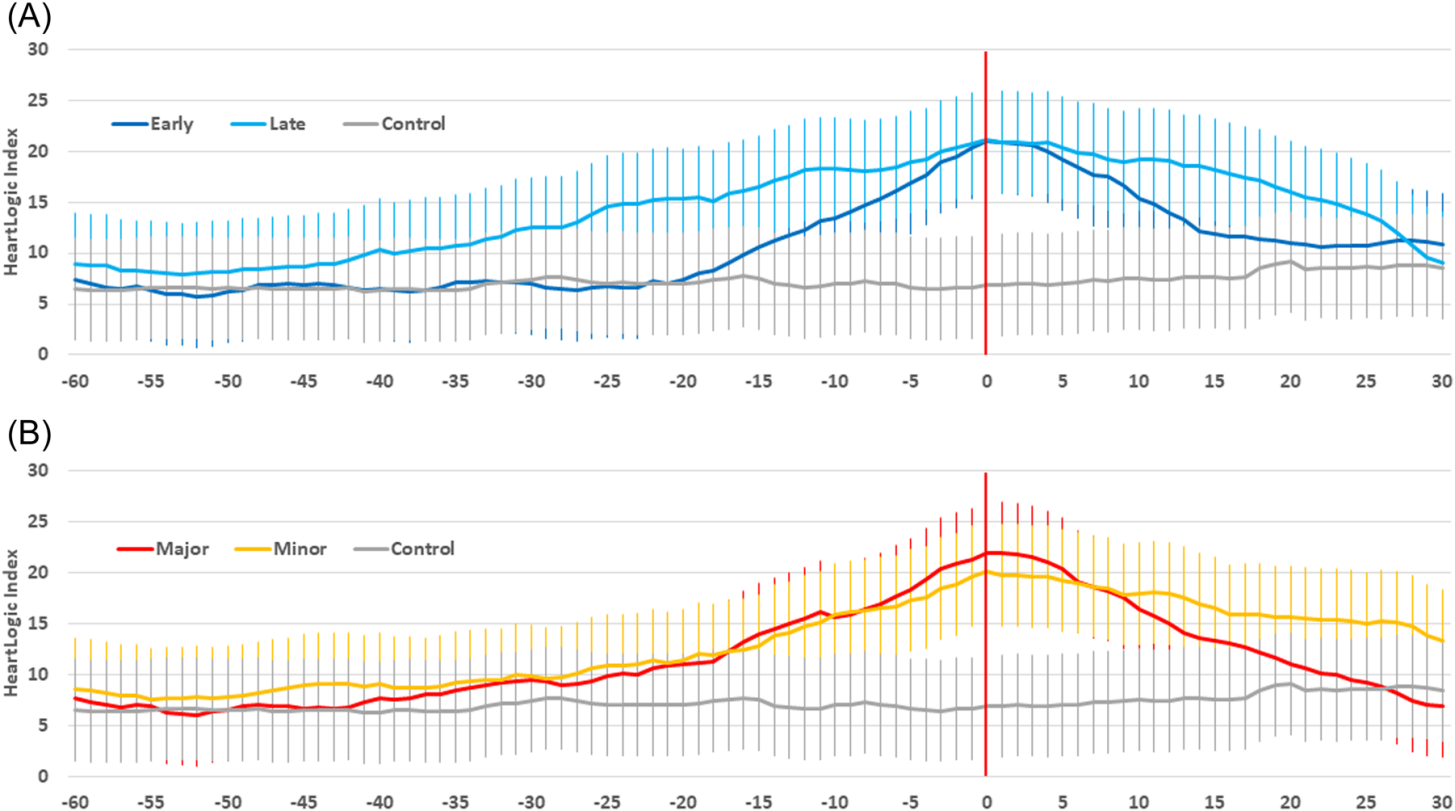
Average HeartLogic index surrounding the decongestive treatment adjustment in the case of: (A) early (*n* = 30) and late (*n* = 26) actions; (B) major (*n* = 29) and minor (*n* = 27) actions. Day 0 is the first day of the diuretic augmentation. Average index from clinically stable periods (*n* = 105, from patients who did not have heart failure events and decongestive treatment adjustments during clinical follow‐up) is reported for comparison

Table 2 shows baseline, preaction, first day of action, and recovery sensor values calculated for the episodes of diuretic adjustments, stratified by time and extent of intervention. In comparison with the baseline period (average calculated 30 days before the intervention), the preaction HeartLogic index value (weekly average calculated 1 day before the intervention) was significantly higher, as was the S3 amplitude. Further worsening occurred on the first day of action in all groups. During the recovery period (14‐day average period beginning 2 weeks postintervention), the HeartLogic combined index and the S3 amplitude improved in all groups, but only when the decongestive treatment adjustment was timely or major did they return to their baseline values. In the control group of clinically stable periods (from patients who did not have HF events and decongestive treatment adjustments during clinical follow‐up) no changes in the combined index or sensor values were noted. The trends in the average HeartLogic index surrounding the decongestive treatment adjustment are reported in Figure 2 with regard to early and late actions, as well as major and minor diuretic augmentations (Day 0 is the first day of the diuretic augmentation). Average data from clinically stable periods are reported for comparison. The trends preceding the intervention were comparable between minor and major treatment adjustments. By contrast, before the action, the HeartLogic index was persistently higher in the case of late diuretic increases than early increases. In the subsequent period, the HeartLogic index decreased after decongestive treatment adjustments, and alert cases resolved more quickly when decongestive therapies were major and timely. The trends in the average sensed parameters that contribute to the calculation of the combined index are reported in Figures S1 and S2.

**Table 2:** Matched sensor data during baseline, preaction, first day of treatment, and recovery, stratified by time (early, late) and extent (major, minor) of intervention

	Baseline (−60 to −30 days)					Preaction (−8 to −1 days)				
	Early	Late	Major	Minor	Control	Early	Late	Major	Minor	Control
HeartLogic index	6.4 ± 6.9	9.3 ± 8.0	7.2 ± 6.6	8.3 ± 8.4	6.6 ± 7.9	17.4 ± 4.8*	19.2 ± 9.6*	19.4 ± 6*	17.7 ± 9.1*	7.0 ± 10.0
S3 amplitude (mG)	1.1 ± 0.4	1.0 ± 0.3	1.1 ± 0.4	1.0 ± 0.3	1.0 ± 0.3	1.2 ± 0.4*	1.1 ± 0.4*	1.2 ± 0.5*	1.1 ± 0.3*	1.0 ± 0.3
S1 amplitude (mG)	2.4 ± 1.1	2.3 ± 0.8	2.5 ± 1.1	2.2 ± 0.8	2.3 ± 1.0	2.4 ± 1.1	2.2 ± 0.8	2.4 ± 1.0	2.2 ± 0.9	2.3 ± 1.0
Thoracic impedance (Ohm)	47 ± 10	44 ± 10	46 ± 10	45 ± 10	49 ± 11	45 ± 9*	44 ± 11	45 ± 10	43 ± 10	48 ± 11
Respiratory rate (breath/min)	18 ± 2	18 ± 2	18 ± 2	18 ± 3	17 ± 2	18 ± 3	18 ± 2	18 ± 2	18 ± 3	17 ± 2
Night heart rate (beats/min)	69 ± 10	67 ± 10	68 ± 11	68 ± 9	68 ± 9	69 ± 9	67 ± 10	67 ± 10	69 ± 8	68 ± 9
Activity (min)	99 ± 53	94 ± 51	96 ± 49	97 ± 54	108 ± 60	96 ± 57	88 ± 51	95 ± 53	89 ± 55	106 ± 59

	**First day of decongestive treatment adjustment**					**Recovery (+14 to** + **28 days)**				
	**Early**	**Late**	**Major**	**Minor**	**Control**	**Early**	**Late**	**Major**	**Minor**	**Control**
HeartLogic index	21 ± 4.7*	21.1 ± 8.1*	22.1 ± 6.3*	20.1 ± 6.5*	6.9 ± 9.3	10.9 ± 13.1	15.3 ± 10.0*	10.8 ± 8.3	15.4 ± 14.4*	8.2 ± 10.6
S3 amplitude (mG)	1.2 ± 0.5*	1.1 ± 0.4*	1.2 ± 0.5*	1.2 ± 0.4*	1.0 ± 0.3	1.1 ± 0.4	1.1 ± 0.3*	1.1 ± 0.4	1.1 ± 0.3*	1.0 ± 0.3
S1 amplitude (mG)	2.4 ± 1.1	2.1 ± 0.7	2.4 ± 1.1	2.1 ± 0.8	2.3 ± 1.0	2.4 ± 1.1	2.1 ± 0.8	2.4 ± 1.1	2.1 ± 0.8	2.3 ± 1.0
Thoracic impedance (Ohm)	44 ± 8*	43 ± 11	45 ± 9	43 ± 10*	48 ± 11	45 ± 9*	45 ± 10	45 ± 10	45 ± 9	47 ± 10
Respiratory rate (breath/min)	18 ± 3	18 ± 3	18 ± 3	18 ± 3	18 ± 2	18 ± 3	19 ± 2	18 ± 2	19 ± 3	18 ± 2
Night heart rate (beats/min)	68 ± 9	67 ± 11	67 ± 10	69 ± 10	68 ± 10	69 ± 7	67 ± 9	67 ± 9	69 ± 8	67 ± 8
Activity (min)	105 ± 68	85 ± 51	106 ± 66	87 ± 57	110 ± 68	100 ± 58	91 ± 49	96 ± 52	94 ± 54	103 ± 60

*Note*: Control group (clinically stable periods from patients with no HF events or decongestive treatment adjustments). Early (*n* = 30), late (*n* = 26), major (*n* = 29), minor (*n* = 27), control (*n* = 105).

* *p* < .05 versus baseline.

### 3.2 Outcome of alert‐triggered actions

**Figure 3:**
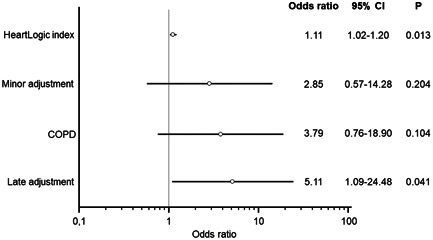
Multivariate analysis. The need for further treatment to resolve the alert condition was associated both with a higher HeartLogic index on the day of initiation of the decongestive treatment adjustment and with late intervention

Overall, timely diuretic changes were associated with a shorter “in‐alert” state duration in comparison with late changes, that is, 28 days (25th–75th percentile: 20–43) versus 62 days (25th–75th percentile: 44–118), *p* < .001. By contrast, major and minor diuretic augmentations resulted in comparable durations, that is, 47 days (25th–75th percentile: 30–58) versus 38 days (25th–75th percentile: 23–79), *p* = .954. Of the 56 decongestive treatment adjustments, 47 (84%) resolved the alert condition, while in the remaining nine cases, further treatments were required (augmented HF therapy during hospitalization or unscheduled intravenous decongestive therapy in outpatients). The need for further treatments was lower after early diuretic adjustments, that is, 1 out of 30 (3%) versus 8 out of 26 (31%, *p* = .008) late adjustments, as well as after major diuretic augmentations, that is, 2 out of 29 (7%) versus 7 out of 27 (26%, *p* = .073) minor augmentations. The need for further treatment to resolve the alert condition was associated both with a higher value of the HeartLogic index on the day of initiation of the decongestive treatment adjustment and with late intervention, in a regression model adjusted for those variables that showed an association on univariate analysis (minor diuretic augmentation and chronic obstructive pulmonary disease) (Figure 3). No complications (e.g., worsening renal function) were reported after drug adjustments. Analysis of the 81 alerts that triggered mixed actions to treat HF showed that, when only one action was required, the alert condition was resolved more rapidly, that is, 45 days (25th–75th percentile: 25–57) versus 60 days (25th–75th percentile: 42–84), *p* = .034, and the need for hospital admission for further treatments was lower, that is, 2 out of 54 (4%) versus 5 out of 27 (19%), *p* = .038.

## 4 DISCUSSION

In this study, we recorded the implementation of the HeartLogic algorithm in the protocol for the remote monitoring of HF patients, and analyzed the clinical actions performed to manage the events of impending HF decompensation notified by the algorithm. We found that the decongestive treatment adjustments triggered by the device alerts were safe and efficacious, and noted that earlier and greater increases in diuretic therapy were associated with more favorable outcomes.

The Multisensor Chronic Evaluation in Ambulatory Heart Failure Patients (MultiSENSE) study ^4^ demonstrated the ability of the HeartLogic algorithm, which combines data from multiple ICD sensors, to reliably predict impending HF decompensation. A further analysis by Gardner et al.^13^ revealed that dynamic assessment by means of the algorithm could identify time‐intervals when patients were at significantly increased risk of worsening HF. In clinical practice, HeartLogic alerts have frequently proved to be relevant and actionable, the rate of alerts judged non‐clinically meaningful, and the rate of HF hospitalizations not associated with alerts being low.^8^, ^9^, ^14^ Moreover, an alert‐based management strategy seems more efficient than scheduled follow‐up schemes. Recent data have also suggested that activation of the multi‐sensor algorithm might result in a significant reduction in hospitalizations for decompensated HF.^15^

Recently, Calò et al.^9^ analyzed the alert management strategies adopted at 22 centers and found that, when clinical actions were undertaken in response to alerts, the rate of HF events was lower. In their experience, actions consisted mainly of drug adjustments. This finding was similar to that of the first experience of the use of HeartLogic in clinical practice,^8^ in which the most frequently reported action triggered by HeartLogic alerts was an increase in diuretic dosage. Also in the present analysis, most post‐alert actions included decongestive treatment augmentations, which were implemented either alone or in combination with other actions (e.g., nondiuretic medication changes, patient education, device reprogramming). Nonetheless, the significant variability of actions taken is encouraging, as it suggests the ability to act on multiple precipitating factors that trigger decompensation of chronic HF (e.g., nonadherence with drugs/diet, new‐onset arrhythmias, loss of CRT, suboptimal device programming, worsening of concomitant comorbidities). For the aim of the present analysis, we specifically investigated isolated increases in the dose of diuretics, to limit confounding factors.

Confirming previous findings,^12^ we showed that, at the time of device detection of the HF event, the sensed parameters that contribute to the calculation of the HeartLogic index presented changes from their baseline reference values. In the present analysis, the accelerometer‐based third heart sound, a surrogate of filling pressure,^16^ significantly increased, as did the respiratory rate, which is known to facilitate the identification of patients at risk of worsening HF.^17^ Similarly, night heart rate, a measure of the patient's autonomic tone,^18^ was higher at the time of the alert.

Our analysis of the recovery period showed that augmenting decongestive treatment in response to an alert was effective in resolving the alert condition. In our study, we categorized diuretic changes as either early or late and as either major or minor. Current recommendations on acute decompensated HF do not mandate specific diuretic increases, and suggest adjusting the dose and duration according to patients' symptoms and clinical status, while at the same time closely monitoring the patient to prevent possible adverse consequences of high‐dose diuretic use.^1^ When automated diagnostics are used for the remote management of HF, centers should intervene in response to alerts long before the onset of acute decompensation, if possible when the patient is still asymptomatic,^8^ to prevent further worsening. Our results suggest that the early use of decongestive treatment (within 2 weeks of the first alert notification) or the use of high doses allows the HeartLogic index and sensor values to return to their baseline values within a month after the intervention. In the case of late treatments or when low doses were used, we noted improvements, but not full recovery. Our analysis of the outcome of alert‐triggered actions confirmed the analysis of trends in sensor values. Indeed, the early use of decongestive treatment was associated with shorter alert duration and fewer hospital accesses for further treatments. The need for further treatment to resolve the alert condition was also associated with a higher value of the HeartLogic index on the day of the treatment adjustment, that is, a more advanced stage at the time of the clinical decision. This is also supported by our additional analysis of those alerts that triggered not only decongestive actions to treat HF. This showed slower resolution of the alert condition and lower efficacy when multiple treatments were needed. The Index trends preceding the intervention were different between early and late treatment adjustments, with the latter increasing more gradually. This is plausibly determined by the greater variability of the intervention time, and its effect on the calculation of the average trend for the late action group. However, we cannot exclude potential differences between groups that might explain the different outcome. Nonetheless, our regression analysis confirmed the association between the time of treatment and the need for further treatments after adjustment for potential clinical confounders.

The present results confirm previous findings from the diuretic optimization strategies evaluation (DOSE) study, which evaluated the optimal approach to diuretic dosing for patients with acute decompensated HF^11^ by randomizing patients to either “low‐dose” or “high‐dose” furosemide. In that study, the high‐dose group had more favorable outcomes, that is, functional class, weight change, net fluid loss, and clinical outcomes. Although high doses of diuretics, which stimulate the renin–angiotensin–aldosterone and sympathetic nervous systems, have been associated with poor outcomes,^19^, ^20^ no adverse consequences of high‐dose diuretic use were reported in either the DOSE study or in our analysis, providing reassurance concerning the safety and utility of this approach in HF.

Previous studies showed that adjusting medications in response to elevated filling pressure values transmitted by an implanted device reduced hospitalizations more effectively than therapy guided only by clinical signs and symptoms of congestion,^21^ and that a greater frequency of therapeutic interventions was linked to a greater reduction in HF events.^22^ Similarly, in our study, interventions were undertaken when multiple physiological sensors identified worsening HF before overt worsening of clinical symptoms. Verification of our observations would require a prospective controlled design, such as that of the ongoing Multiple Cardiac Sensors for the Management of Heart Failure (MANAGE‐HF) trial (NCT03237858). Even more importantly, it would require a standardized treatment algorithm, to encourage clinicians to take prompt and major actions in response to presymptomatic alerts. This would probably prevent the failure in reducing clinical events that has been previously reported when telemedicine alerts were not handled appropriately. Indeed, in the Optimization of Heart Failure Management Using OptiVol™ Fluid Status Monitoring and CareLink™ (OptiLink HF trial), impedance‐based remote monitoring failed to reduce clinical events,^23^ the failure being ascribed to the low rate of alert‐driven interventions. Another reason could be the low sensitivity of impedance‐based HF alerts^24^ that may have resulted in not treating potentially actionable events, or the high rate of false‐positive detections and a consequent increase in hospital admissions.^25^ The adoption of a multi‐parameter algorithm, that has proved to be sensitive and associated with a low rate of unexplained detections,^4^ should overcome these limitations.

### 4.1 Limitations

The main limitation of this study is its observational non‐randomized design. Although the observational nature of the analysis may have introduced some biases, the consecutive enrollment should have decreased their magnitude. Indeed, the assignment of a HeartLogic‐enabled device was not guided by the characteristics of the patients. Moreover, no predetermined actions were prescribed in response to HeartLogic alerts or to the individual subject's reported signs or symptoms. However, owing to the “real‐world” nature of the study, this could even strengthen the usefulness of HeartLogic as a viable monitoring tool in everyday clinical practice.

## 5 CONCLUSIONS

This study demonstrated the safety and efficacy of decongestive treatment adjustments triggered by HeartLogic alerts, even when such adjustments were completely dependent on the physicians' clinical expertise and were not standardized. Our results suggest that the early use of decongestive treatment and the use of high doses of diuretics are associated with more favorable outcomes.

## CONFLICTS OF INTEREST

M. Campari and S. Valsecchi are employees of Boston Scientific. The other authors declare no conflicts of interest.

## Supporting information


**Supplemental Figure 1**. Average sensor values surrounding the decongestive treatment adjustment in the case of early (*n *= 30) and late (*n* = 26) actions (Day 0 is the first day of the diuretic augmentation). Average sensor data from clinically stable periods (*n *= 105, from patients who did not have heart failure events and decongestive treatment adjustments during clinical follow‐up) are reported for comparison.

**Supplemental Figure 2**. Average sensor values surrounding the decongestive treatment adjustment in the case of major (*n *= 29) and minor (*n *= 27) actions (Day 0 is the first day of the diuretic augmentation).

## Data Availability

The experimental data used to support the findings of this study are available from the corresponding author upon request.

## 1

Full list of participant centers and investigators

- Policlinico Casilino, Rome, Italy: Calò L., De Ruvo E., Minati M., Tota C., Martino A.
- Monaldi Hospital, Naples, Italy: D'Onofrio A., Bianchi V., Tavoletta V.
- OO. RR. San Giovanni di Dio Ruggi d'Aragona, Salerno, Italy: Ferraioli D., Manzo M.
- “Giovan Battista Grassi” Hospital, Rome, Italy: Santini L., Ammirati F., Mahfouz K., Colaiaco C.
- Università Politecnica delle Marche, “Ospedali Riuniti”, Ancona, Italy: Dello Russo A., Guerra F.
- Fondazione Poliambulanza, Brescia, Italy: La Greca C., Pecora D.
- IRCCS Policlinico San Matteo, Pavia, Italy: Petracci B.
- “Spaziani” Hospital, Frosinone, Italy: Giubilato G., Carbonardi L.
