# Supplementary figures and images for: Decongestive treatment adjustments in heart failure patients remotely monitored with a multiparametric implantable defibrillators algorithm
Source: Clin Cardiol. 2022 May 3;45(6):670–8. doi: 10.1002/clc.23832 (PMC9175259; doi:10.1002/clc.23832)

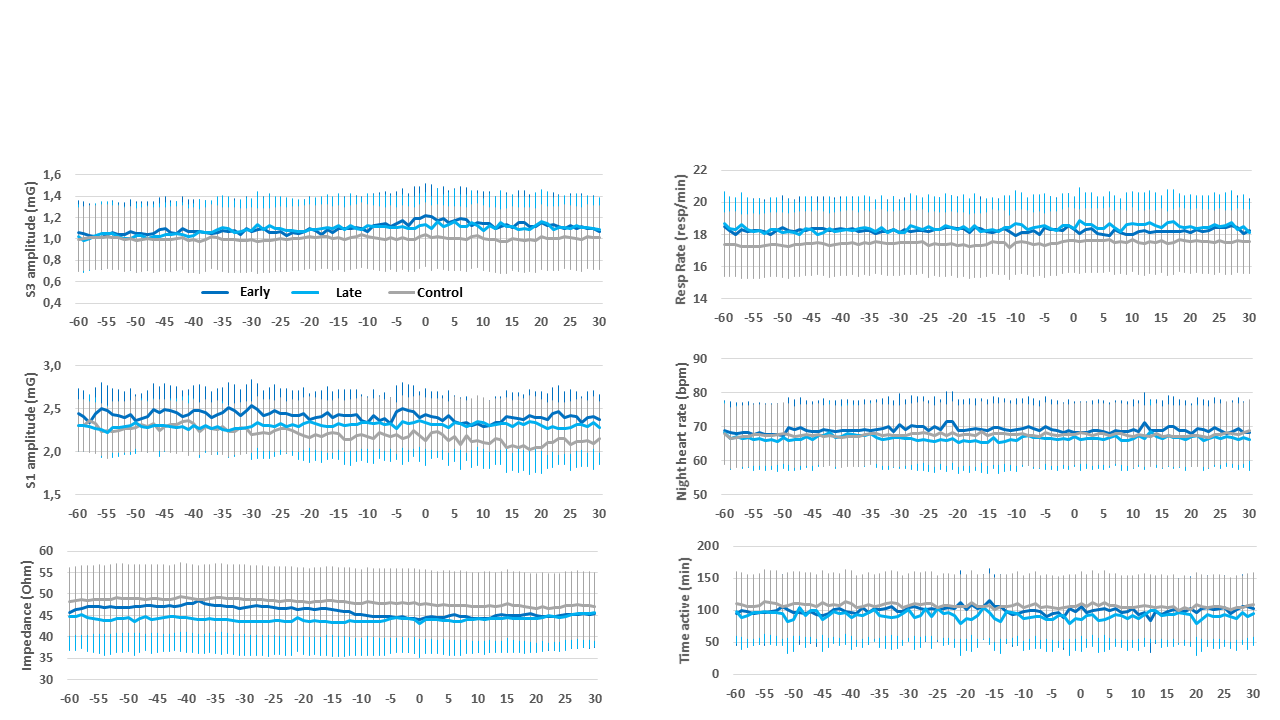

Supplement: Supplementary file 1 — Supplemental Figure 1. Average sensor values surrounding the decongestive treatment adjustment in the case of early (n = 30) and late (n = 26) actions (Day 0 is the first day of the diuretic augmentation). Average sensor data from clinically stable periods (n = 105, from patients who did not have heart failure events and decongestive treatment adjustments during clinical follow‐up) are reported for comparison. [file CLC-45-670-s001.tif]

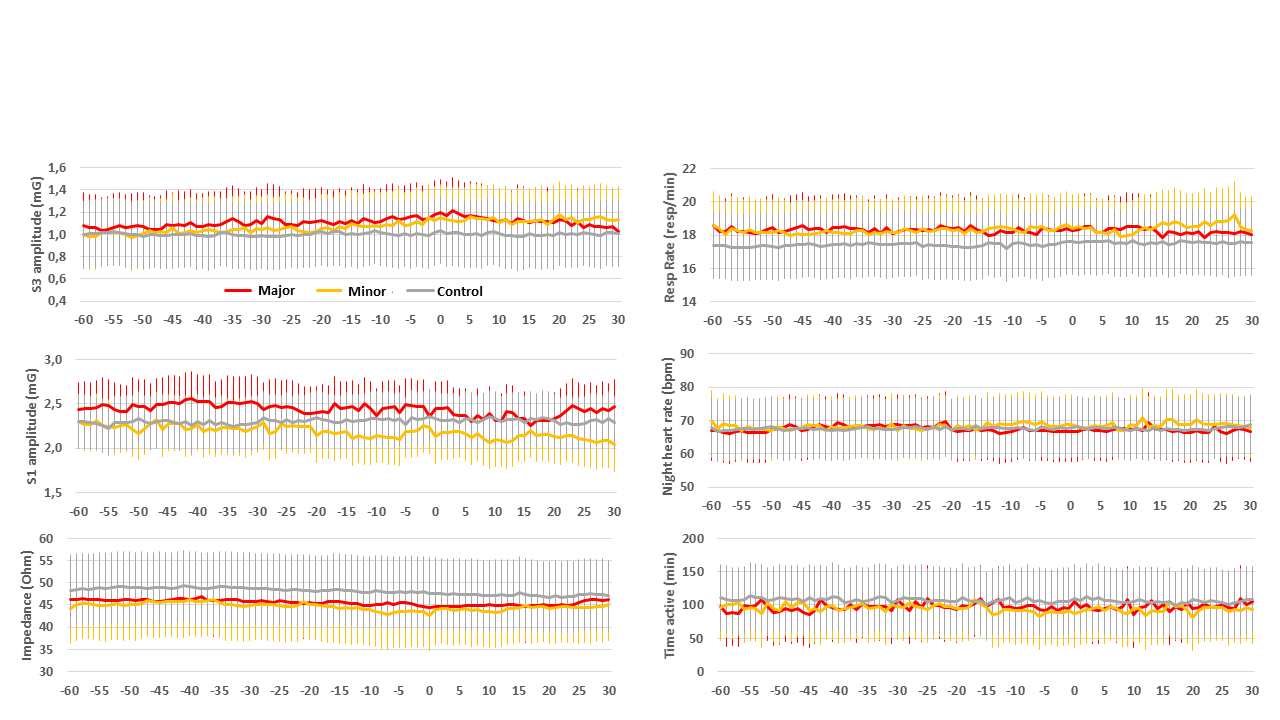

Supplement: Supplementary file 2 — Supplemental Figure 2. Average sensor values surrounding the decongestive treatment adjustment in the case of major (n = 29) and minor (n = 27) actions (Day 0 is the first day of the diuretic augmentation). [file CLC-45-670-s002.tif]
